# Effect of Steaming and Microwave Heating on Taste of Clear Soup with Split-Gill Mushroom Powder

**DOI:** 10.3390/foods12081685

**Published:** 2023-04-18

**Authors:** Vimolpa Hiranpradith, Nantawan Therdthai, Aussama Soontrunnarudrungsri

**Affiliations:** Department of Product Development, Faculty of Agro-Industry, Kasetsart University, Bangkok 10900, Thailand

**Keywords:** umami, salt, E-tongue, 5′-nucleotides, salinity, free amino acids

## Abstract

Salt is widely overconsumed. Among the strategies used in low-salt foods, the addition of flavor enhancers to improve saltiness perception through an umami taste is a viable and promising technique. This study investigated using split-gill mushroom (SGM) powder containing umami taste to increase saltiness in a clear soup for two different heating conditions: steaming under high pressure and microwave heating. According to the E-tongue results, the addition of 0.2–0.8% SGM produced a different taste in the soup compared to the addition of salt, and the addition of 0.2–0.8% SGM yielded a similar taste to the addition of 0.4–0.6% MSG in a plain, clear soup. In flavored soup, SGM at a high concentration had a taste-enhancing impact comparable to 0.4% MSG, whereas SGM at a low concentration had no taste-enhancing effect. The flavored soups containing 0.4 or 0.8% SGM consisted of two umami 5′-nucleotides: adenosine 5′-monophosphate (5′-AMP) and guanosine 5′-monophosphate (5′-GMP); however, inosine 5′-monophosphate (5′-IMP) was not detected. The major umami amino acids were glutamic acid, aspartic acid, and arginine. Microwave heating increased the salinity and total nucleotides and could maintain the umami amino acids, whereas aspartic acid (one of the umami amino acids) was reduced by 8.23% after steaming under high pressure. Thus, after microwave heating and steaming under high pressure, the equivalent umami concentration was reduced by 43.11 and 44.53%, respectively. In conclusion, the addition of SGM and volumetric heating using microwaves could be an alternative method for reducing the amount of salt in soup by increasing the umami taste intensity and salinity.

## 1. Introduction

Salt (NaCl) is regarded as the king of all flavors and is used by most cuisines to enhance saltiness and the savory flavor. However, salt is overconsumed widely due to its major impact on the palatability and consumer acceptance of a food item [1]. The World Health Organization (WHO) advises adults to consume less than 5 g of salt daily to reduce their risk of developing hypertension and cardiovascular diseases [2]. However, studies from several nations suggested that most populations consumed around 9–12 g of dietary salt per day on average, which is 2–2.5 times higher than recommended [2]. It is projected that lowering salt consumption to the recommended amount would improve public health globally and avoid about 2.5 million deaths from non-communicable diseases (NCDs) each year [2]. There are difficulties for the food industry in lowering salt in recipes, because this affects not only taste but also mouthfeel and the microbiological safety of food. Inorganic salts, such as potassium chloride (KCl) and calcium chloride (CaCl_2_), have been proposed as alternatives to sodium chloride (NaCl) in order to reduce salt over the past few decades. The disadvantage of this strategy is that it results in a bitter, metallic taste, which has a negative impact on consumer acceptance and limits the use of inorganic salts as replacements [3,4]. Another option is to use the stealth method, which involves gradually reducing the concentration of salt in food in a way that consumers cannot notice; however, this can be inefficient and time consuming [5]. Among the strategies used in low-salt foods, the inclusion of flavor enhancers to enhance saltiness perception through umami taste is a viable technique with high potential [1,6]. Monosodium glutamate (MSG) is an acid salt of glutamic acid that has been used as a flavor enhancer in the food industry for over a century. Despite the fact that the FAO and the WHO have declared it to be safe, a study found that more than 60% of people in the United States avoided or reduced their consumption of MSG-containing foods [7]. In addition, Selani et al. [8] discovered that consumers rated burgers containing MSG as unhealthy, artificial, and additive, compared to burgers containing mushroom-flavored enhancer, which were rated positively. Given the widespread disapproval of MSG use, using mushrooms as a natural flavor enhancer to minimize salt in meals could satiate customers’ cravings for savory and healthy foods [9,10,11].

Mushrooms are fungi that can be found all over the world. They are rich in protein, low in fat, high in dietary fiber, and additionally, they have a distinct scent and are a great source of umami compounds, such as free amino acids and 5′-nucleotides [12]. Many studies have been conducted on the use of mushroom extract as a replacement for MSG and its ability to reduce sodium in various food types. For example, the sensory evaluation of 99 Brazilian consumers of low-sodium corn-extruded snacks revealed that flavor enhancers derived from shiitake byproducts had a salty and umami taste similar to MSG [13]. Guinard et al. [14] investigated consumer acceptance of taco blends containing white mushrooms instead of beef that had 25% less salt. According to their findings, the mushrooms could be used to replace beef while lowering sodium levels without affecting consumer liking. Split-gill mushroom (*Schizophyllan commune* spp.) is one of Thailand’s most cultivated native mushrooms [15]. It contains *Schizophyllan*, a unique glucan with anti-inflammatory and immune-boosting properties [16,17,18]. Although mushrooms have a high content of umami amino acids, thermal processing during cooking may lessen their concentration. According to Li et al. [19], microwave cooking yielded the highest umami content, followed by boiling and autoclaving, respectively. Contrarily, Sun et al. [20] found that compared to autoclaving, steaming, and microwave cooking, sous vide produced the most 5′-nucleotides overall, with microwave cooking producing the least glutamic and aspartic acids. Additionally, Zhang et al. [21] concluded that drying and cooking had an effect on the guanylate content of mushrooms.

Soup is one of the most sodium-rich foods, with several studies observing that commercial soups marketed all over the world often contain excessive quantities of salt, ranging from 0.5% to more than 1%, posing a serious threat to global health [22,23,24,25]. Based on the authors’ knowledge, few studies have been conducted using the split-gill mushroom and its potential use as a flavor enhancer to reduce sodium content in soup. 

Therefore, the purpose of this research was to investigate the effect of different concentrations of split-gill mushroom powder on the taste profile of clear soup using an E-tongue and to investigate the effect of heating on the amounts and types of taste compounds in clear soup containing split-gill mushroom powder. The findings relating to the taste profile of split-gill mushroom powder and the effect of heating procedures should play an important instructive role in flavor design during the development of food items and cuisine with reduced salt, potentially creating a new additional value for split-gill mushroom powder as a perception enhancer of saltiness.

## 2. Materials and Methods

### 2.1. Materials

#### 2.1.1. Standards and Reagents

Analytical-grade standards for the chemical analysis of samples, such as 0.1 N silver nitrate standard solution (Pine Chemical™, Nonthaburi, Thailand), potassium chloride (Daejung™, Gyeonggi-do, Republic of Korea), and potassium chromate (KemAus™, Cherrybrook, NSW, Australia), were purchased from local vendors. Cytidine 5′-monophosphate disodium salt (5′-CMP, ≥99%; AR grade), uridine 5′-monophosphate disodium salt (5′-UMP, ≥99%; AR grade), and L-glutamic acid monosodium salt were purchased from Alfa Aesar™ (Heysham, UK). Guanosine 5′-monophosphate disodium salt hydrate (5′-GMP, ≥97%; AR grade), methanol (HPLC grade), and ultrapure water (HPLC grade) were purchased from Fisher Scientific™ (Loughborough, UK). Orthophosphoric acid (HPLC grade) was purchased from Loba Chemie™ (Tarapur, India). Inosine 5′-monophosphate disodium salt hydrate (5′-IMP, ≥99.0%; HPLC grade) and adenosine 5′-monophosphate disodium salt (5′-AMP, ≥99.0%; HPLC grade) were purchased from Sigma–Aldrich Co., Ltd. (Singapore).

#### 2.1.2. Ingredients

Dried split-gill mushroom (SGM) powder was purchased from Chaiyo farm (Suratthani, Thailand). Dried onion powder, garlic powder, ground black pepper, and Bay leaves were purchased from Nguansoon, Thailand. Dried carrot powder (Gosenga™, Bangkok, Thailand) was purchased from a local provider in Thailand. Monosodium glutamate (Ajinomoto™, Bangkok, Thailand; MSG), sugar, and salt were also purchased from a local provider in Thailand.

### 2.2. Sample Preparation

#### 2.2.1. Clear Soup (without Seasoning and Spices) Preparation for Analysis with E-Tongue

**Figure 1:**
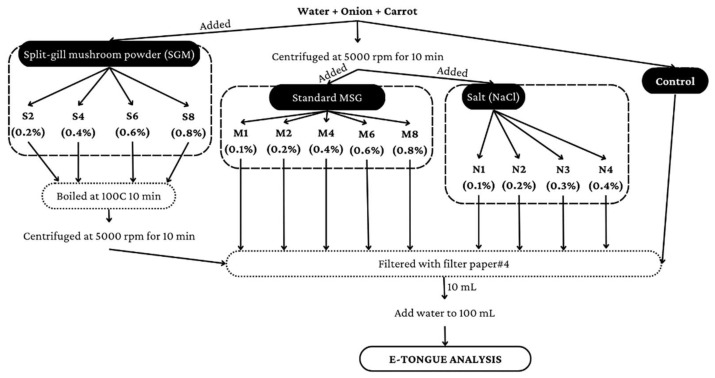
Preparation process of clear soup (without seasoning and spices) for E-tongue analysis.

Water (1.5 L) was mixed with an onion (10 g) and carrot powder (7.5 g). Then, they were all boiled at 120 °C for 13 min, after which the soup temperature was maintained at 100 ± 5 °C for 32 min. Following that, the soup was strained to remove residues and allowed to cool at room temperature. Samples (each 500 mL) of the cooled soup were packed in PE bags and frozen at −20 °C for further analysis by E-tongue (Figure 1).

#### 2.2.2. Clear Soup (with Seasoning and Spices) Preparation for E-Tongue Analysis

**Figure 2:**
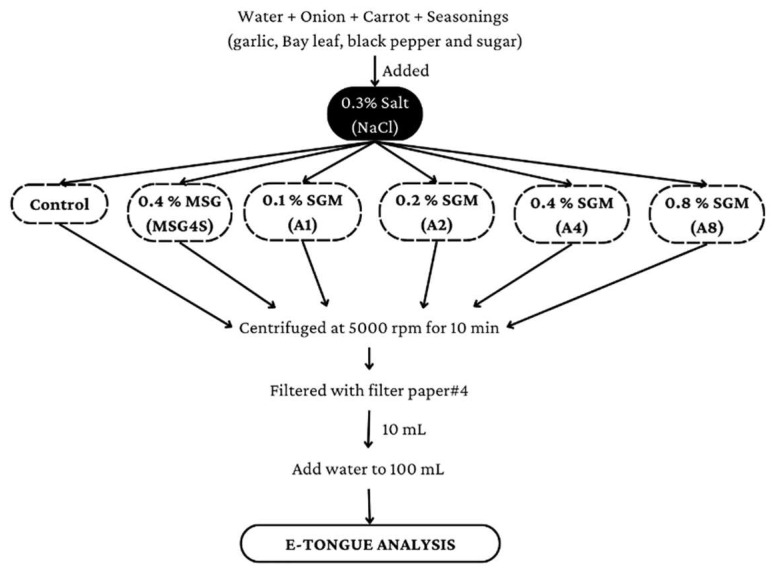
Preparation process of flavored clear soup for E-tongue analysis.

Water (1.5 L) was mixed with an onion (10 g) and carrot powder (7.5 g), 0.4 g garlic powder, 1 Bay leaf, 0.5 g black pepper powder, 2 g sugar, and other ingredients as shown in Figure 2. The preparation procedure was as described in Section 2.2.1. Each sample (500 mL) was packed in a PE bag and frozen at −20 °C for E-tongue analysis within the following few days.

#### 2.2.3. Clear Soup (with Seasoning and Spices) Preparation for Chemical Analysis

**Figure 3:**
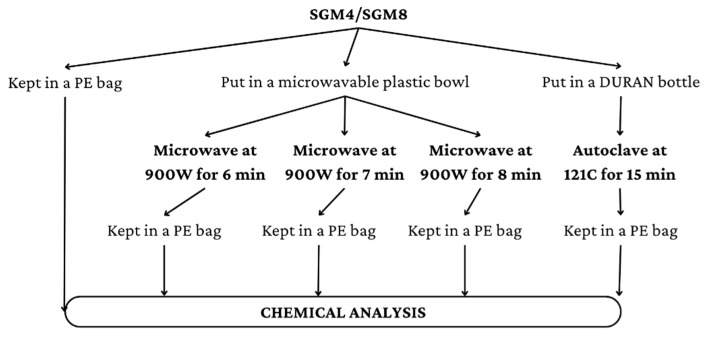
Microwave heating and steaming processes of SGM4 and SGM8 before chemical analysis.

For each sample, water (1.5 L) was mixed with 0.4% SGM powder (SGM4), 0.8% SGM powder (SGM8), and the other ingredients, as listed in Figure 2 (except for the NaCl, which was reduced to 0.2%), and placed on an electronic stove to boil at 120 °C for 13 min, after which the soup temperature was maintained at 100 ± 5 °C for 32 min. Then, the soup was strained to remove any remaining residues and allowed to cool at room temperature. Then, samples were equally divided into five portions. The first portion was frozen at −20 °C in a PE bag for further analysis. Other portions were further heated using a microwave (LG MP-9482S output 2450 MHz) at 900 W for 6, 7, or 8 min or steaming under high pressure in an autoclave (Hirayama) for 15 min. The additional heating processes are illustrated in Figure 3. After that, all samples were packed in separate PE bags and frozen at −20 °C.

### 2.3. E-Tongue Analysis

An E-tongue α-ASTREE (Alpha MOS Company, Toulouse, France) was used to determine the taste correlations between SGM and L-glutamic acid monosodium salt or salt (NaCl) at different concentrations in a clear soup model. The E-tongue included a sensor array, an automatic sampler, a special beaker, a signal acquisition and processing system, and a data analysis software package. Six different sensors (AHS, CTS, NMS, ANS, SCS, PKS, and CPS) were used to detect sourness, saltiness, umami, sweetness, bitterness, and other water-soluble compounds in samples.

The sample analysis was divided into two tests. The first test was to detect any flavor correlations in the clear soup (without seasoning or spices) between SGM and L-glutamic acid monosodium salt or salt (NaCl) at various concentrations by dividing the prepared clear soup into 14 portions. Nine of the fourteen portions were centrifuged at 5000 rpm for 10 min before separating the supernatants. After that, to maintain the concentrations specified in the protocols, the supernatants from the first five portions were treated with L-glutamic acid monosodium salt at concentrations of 0.1, 0.2, 0.4, 0.6 and 0.8%. (M1–M8). Others were given NaCl concentrations of 0.1, 0.2, 0.3 and 0.4% (N1–N4). Then, one of the remaining five components from the clear soup (without seasoning, spices, or SGM) was kept as a control. Then, the remaining four parts of clear soup (without seasoning or spices) were blended with 0.2, 0.4, 0.6 or 0.8% SGM before being cooked at 100 °C for 10 min to extract taste compounds (S2–S8). The clear soups containing SGM were centrifuged for 10 min at 5000 rpm. To obtain a clean solution, the supernatant from all samples was filtered through filter paper #4. Each sample solution (10 mL) was diluted with water to a volume of 100 mL. Figure 1 depicts the preparation process of the clear soup (without seasoning or spices), as modified from [26]. The second test was to detect any correlations in taste between SGM and MSG or salt (NaCl) at various concentrations in 0.3% salted-clear soup with seasoning and spices as a standard recipe. Aside from 0.3% salt, no MSG or SGM were introduced to the control salted-clear soup. All clear soup samples were centrifuged and filtered, as described previously in Figure 2. 

For E-tongue analysis, 80 mL of each sample were used. Each collection time was set at 120 s, and one measurement was obtained per second [27]. Each sample was prepared in six batches and measured in a row. Data were exported, and outlier data were discarded before analysis of the remaining data. Principal component analysis (PCA) using correlation and cluster analysis at the 95% confidence level were performed using the XLSTAT statistical software for Microsoft Excel.

### 2.4. Determination of Moisture, Protein, and Sodium Contents

All measurements were taken in triplicate, according to the procedure of the Association of Official Analytical Chemists (AOAC) [28]. The moisture content was determined using AOAC Method 930.15 by removing moisture in a hot-air oven (Binder FD115) at 105 °C from each sample to a constant weight. The crude protein content in the samples was determined using the Kjeldahl method. For calculation, total nitrogen was multiplied by 6.25, according to AOAC Method 954.01. Salinity was determined using a refractometer (ATAGO S-28E). The percentage of NaCl was determined using the AOAC Method 960.29. Samples (2 g) were placed in a crucible and heated in a furnace at 450 °C until they turned to ash. The ash from each sample was mixed with 10 mL of deionized water before being placed in an Erlenmeyer flask. Each sample received 1 mL of K_2_CrO_4_ and was titrated with 0.1 M of AgNO_3_. Then, the NaCl content was calculated using Equation (1):

(1) $$\mathrm{NaCl}\;(\%)=\frac{\mathrm{mL}\;\mathrm{of}\;{\mathrm{AgNO}}_{3}\;(\mathrm{Sample}\;-\;\mathrm{Blank})\;\times \;0.0584\;\times \;100\;\times \;\mathrm{mol}\;\mathrm{of}\;{\mathrm{AgNO}}_{3}}{\mathrm{Sample}\;\mathrm{weight}\;(g)}$$

### 2.5. 5′-Nucleotide Assay

The samples were centrifuged for 30 min at 4000 rpm. Prior to HPLC analysis (Agilent 6420), the supernatant was passed through a 0.22 µm nylon filter. The 5′-nucleotides were separated on a Kinetex 2.6 µm EVO C18 (100 × 2.10 mm) column for 15 min at a flow rate of 0.5 mL/min with an isocratic mobile phase of 5% A and 95% B (A: methanol and B: 0.05% phosphoric acid) and UV detection at 254 nm. Each 5′-nucleotide was identified and quantified by comparing its retention time to that of an authentic standard, modified from [26].

### 2.6. Free Amino Acid Assay

Amino acid profiles of samples were analyzed using an amino acid analyzer at Central Laboratory (Bangkok, Thailand) Co., Ltd., based on an in-house method TE-CH-372 and on TE-CH-373 based on the Official Journal of the European Communities, L257/16 (1983) [29,30]. 

### 2.7. Equivalent Umami Concentration (EUC)

The measurement of umami taste is expressed in terms of the EUC [31], which is the amount of MSG present in 100 g of dry food; therefore, the total umami taste can be calculated using Equation (2):

(2) $$Y=\sum a_{i}b_{i}+1218(\sum a_{i}b_{i})(\sum a_{j}b_{j})$$

where a_i_ is the concentration of umami amino acids (aspartic acid: Asp and glutamic acid: Glu), a_j_ is the concentration of umami 5′-nucleotides (5′-AMP, 5′-IMP, 5′-GMP, and 5′-XMP), b_i_ is a constant value for umami amino acids relative to MSG (Glu = 1, Asp = 0.077), b_j_ is a constant value for taste nucleotides relative to that of 5′-IMP (5′-IMP = 1.00, 5′-AMP = 0.18, 5′-GMP = 2.30, 5′-XMP = 0.61), and Y is the equivalent umami concentration (g/100 g) [26].

### 2.8. Statistical Analysis

All experiments were carried out in triplicate, with data presented as mean ± standard deviation and mean rank. The Kruskal–Wallis H test was used with pairwise comparisons, and the Dunn–Bonferroni test was used for multiple comparisons with statistical significance tested at the *p* ≤ 0.05 level. IBM SPSS Statistics 28.0 software (Thaisoftup Co., Ltd., Bangkok, Thailand) was used to calculate all data based on a 95% confidential level.

## 3. Results and Discussion

### 3.1. Taste Profile of Different Concentration of SGM in Clear Soup

**Figure 4:**
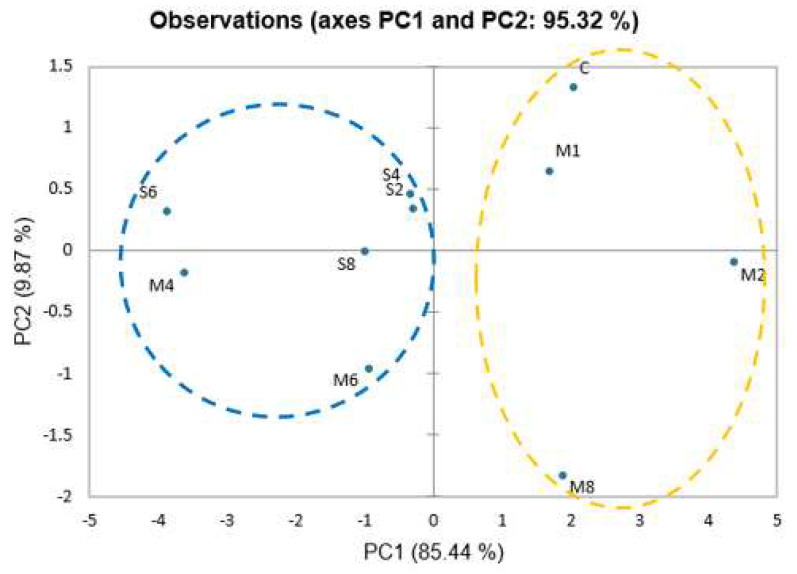
PCA score plots of taste attributes based on E-tongue in control unseasoned soup, unseasoned soup containing 0.2–0.8% SGM, and unseasoned soup containing 0.1–0.8% MSG. All samples were grouped based on cluster analysis and delineated by blue and yellow ovals (*p* ≤ 0.05), where C: control unseasoned soup sample; M1: 0.1% MSG unseasoned soup, M2: 0.2% MSG unseasoned soup, M4: 0.4% MSG unseasoned soup, M6: 0.6% MSG unseasoned soup, M8: 0.8% MSG unseasoned soup, S2: 0.2% SGM unseasoned soup, S4: 0.4% SGM unseasoned soup, S6: 0.6% SGM unseasoned soup, and S8: 0.8% SGM unseasoned soup.

Figure 4 depicts the PCA score plots for the control unseasoned soup and the unseasoned soup with SGM or MSG obtained using the E-tongue. The combined contribution rate of the two main principal components (PC1 and PC2) reached 95.32%, showing that the taste profiles of the different samples could be discriminated efficiently [27]. Cluster analysis revealed that all samples could be divided into two categories. All SGM samples (S2–S8), M4, and M6 were in the same group (delineated by a blue oval in Figure 4), indicating that SGM at all concentrations had a taste characteristic similar to M4 and M6. The control, M1, M2, and M8 were classified into the second group, implying that their tastes were comparable. These findings suggested that unseasoned soup containing 0.2–0.8% SGM had an umami flavor similar to the unseasoned soup containing 0.4–0.6% MSG.

**Figure 5:**
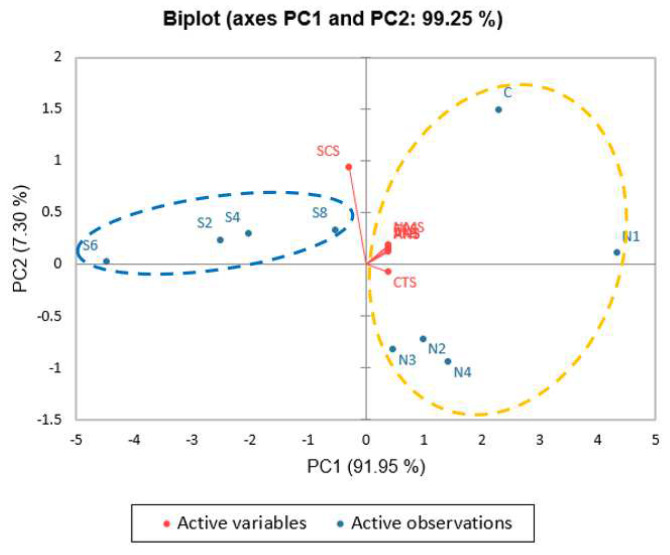
PCA biplot of E-tongue flavor attributes in control unseasoned soup, unseasoned soup with 0.2–0.8% SGM, and unseasoned-clear soups with varying NaCl concentrations. Red dots indicate E-tongue sensors. Differences among samples were identified using cluster analysis, and groups are represented by blue and yellow ovals (*p* ≤ 0.05). C: Control unseasoned soup sample, N1: 0.1% NaCl unseasoned-clear soup, N2: 0.2% NaCl unseasoned-clear soup, N3: 0.3% NaCl unseasoned-clear soup, and N4: 0.4% NaCl unseasoned-clear soup; S2: 0.2% SGM unseasoned soup, S4: 0.4% SGM unseasoned soup, S6: 0.6% SGM unseasoned soup, and S8: 0.8% SGM unseasoned soup.

Figure 5 depicts the PCA, with PC1 and PC2 explaining 91.95% and 7.30%, respectively, of the total variance between unseasoned soups with 0.2–0.8% SGM, unseasoned soups with 0.1–0.4% NaCl and without NaCl. Analysis revealed that the samples could be divided into two groups: (1) SGM-unseasoned soup (S2–S8) and (2) unseasoned soup with NaCl (N1–N4) and the control (C), reflecting that those soups with SGM alone tasted different from the soups with salt. In addition, N2 and N4 were closer to the CTS sensor, which detects salty taste, than any other samples, representing its dominance in the salty taste profile. According to these results, SGM yielded a different salty taste from NaCl in the unseasoned soup.

**Figure 6:**
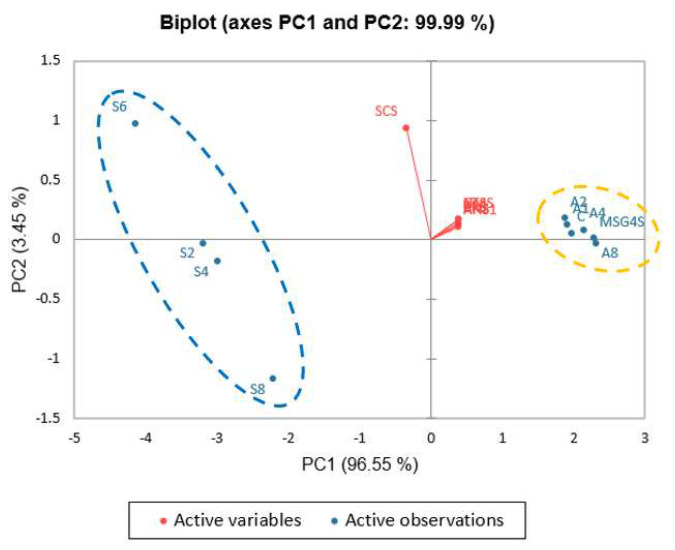
PCA biplot of flavor attributes in unseasoned and standard soups with different SGM concentrations. Differences among samples were identified using cluster analysis, and groups are represented by blue and yellow ovals (*p* ≤ 0.05). C: Control standard soup sample, A1: 0.1% SGM standard soup, A2: 0.2% SGM standard soup, A4: 0.4% SGM standard soup, A8: 0.8% SGM standard soup, MSG4S: 0.4% MSG standard soup, and S1–S8: 0.1–0.8% SGM unseasoned soup, respectively.

**Figure 7:**
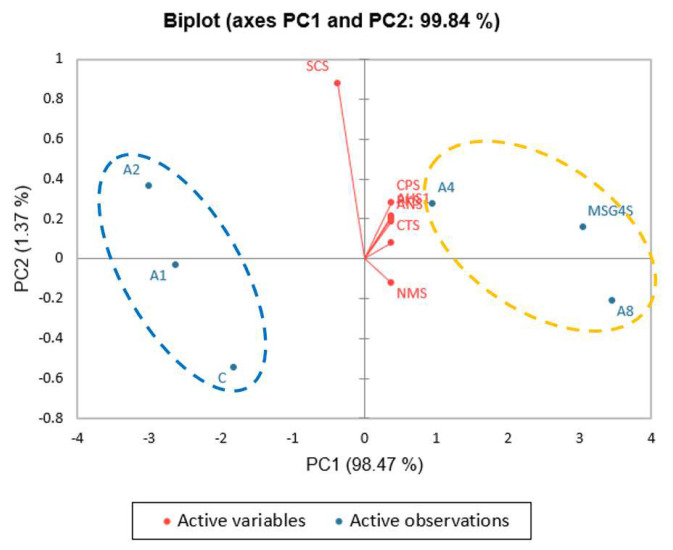
PCA biplot of standard soups with varying concentrations of SGM, standard soup with 0.4% MSG, and control standard soup. Differences among samples were identified using cluster analysis, and groups are represented by blue and yellow ovals (*p* < 0.05). C: Control standard soup sample, A1: 0.1% SGM standard soup, A2: 0.2% SGM standard soup, A4: 0.4% SGM standard soup, A8: 0.8% SGM standard soup, and MSG4S: 0.4% MSG standard soup.

Based on these findings, SGM at 0.2–0.8% concentrations yielded a taste profile comparable to MSG at 0.4–0.6%, whereas their salty profiles were entirely different from that of salt. However, a regular soup recipe for human consumption would include salt, sugar, and other spices to enhance the flavor of the soup; thus, taste profiles from unseasoned-clear soup were compared to those from the standard recipe. The tastes of the unseasoned and standard soups could be clearly distinguished using PCA, showing that seasoning could completely change the taste of the soup. (Figure 6). Furthermore, PCA between the standard clear soups containing varying concentrations of SGM (C, A1, A2, A4, and A8) and 0.4% MSG (MSG4S) revealed that the standard clear soup containing 0.4% MSG tasted comparable to the standard soups containing 0.4% or 0.8% SGM (Figure 7). In addition, A8 was closer to the NMS sensor (which detects umami flavor) than other samples, including MSG4S, signaling that A8 may have had a greater umami taste intensity than MSG at 0.4% in the standard clear soup. 

Researchers [12,13,14,32] discovered that mushrooms can be used to improve the saltiness perception in several food types; likewise, in the current study, the E-tongue was able to discern differences in the tastes of standard soup containing 0.4–0.8% SGM, similar to 0.4% MSG. Furthermore, greater concentrations of SGM were found to be more potent in terms of overall taste, including salty taste, than a low concentration of SGM and the controls. Wang et al. [33] found that umami taste at low concentrations had neither a suppressive nor an enhancing impact on other flavors. Consequently, a low concentration of SGM had no enhancing impact. 

### 3.2. Moisture, Protein, and Sodium Contents and Taste Compounds of Clear Soups 

The E-tongue results showed that SGM at 0.4–0.8% had a flavor similar to MSG at 0.4% in the standard soup. Therefore, a minimum concentration of 0.4% and a maximum concentration of 0.8% were chosen for further investigation using a reduced-salt clear soup model. 

Table 1 shows that the moisture contents of standard clear soup with 0.4% and 0.8% SGM (SGM4 and SGM8) were 98.24% and 98.07%, respectively. SGM4 had a protein content of 18.94%, whereas SGM8 had a protein content of 20.51%. Naturally, higher protein levels should result from SGM8 having more SGM than SGM4. On a wet basis, the NaCl contents in SGM4 and SGM8 were 0.22% and 0.23%, respectively, which was consistent with the amount of NaCl specified in the recipe (0.2%). However, the Na contents measured using salometers were significantly higher than the NaCl contents measured based on a Mohr titration, implying that there was another source of Na (other than salt) that contributed to salinity. According to Mleczek et al. [34], different mushroom species have different mineral concentrations. In addition, between 2009 and 2017, Na was discovered in the fruiting bodies of *Agaricus bisporus*, *Pleurotus ostreatus*, and *Lentinula edodes* at concentrations in the range 67–1440 mg/kg dry weight. Similarly, Zsigmond et al. [35] compared the mineral contents of *Agaricus campestris* mushrooms in urban and peri-urban areas of Transylvania and discovered that their Na content varied depending on the growing areas from 220 to 1700 mg/kg dry weight in mushroom caps and from 205 to 3900 mg/kg in stipes. These reports provided evidence for why the mushroom samples had a higher total Na content than was present in the added NaCl and why SGM8, which contained more SGM, had a slightly higher Na content than SGM4.

The amounts of 5′-nucleotides in SGM4 and SGM8 are shown in Table 2. No 5′-IMP was found in any sample, but 5′-CMP was the most abundant at 1177.94 ± 70.44 mg/100 g (dry weight) and 915.12 ± 54.72 mg/100 g (dry weight) for SGM8 and SGM4, respectively. The highest content of 5′-CMP in various mushrooms was also found in other studies [36,37,38]. The second-most abundant compound found in the soup samples was 5′-UMP, with 229.98 ± 23.39 mg/100 g (dry weight) for SGM4 and 124.46 ± 12.66 mg/100 g (dry weight) for SGM8. Despite their abundance, 5′-CMP and 5′-UMP have no taste attributes [39]. The umami nucleotides, including 5′-AMP and 5′-GMP, were discovered in trace amounts in both SGM4 and SGM8.

### 3.3. Effect of Microwave Heating and Steaming under Pressure on Protein and Sodium Contents and Taste Compounds of Clear Soups 

**Figure 8:**
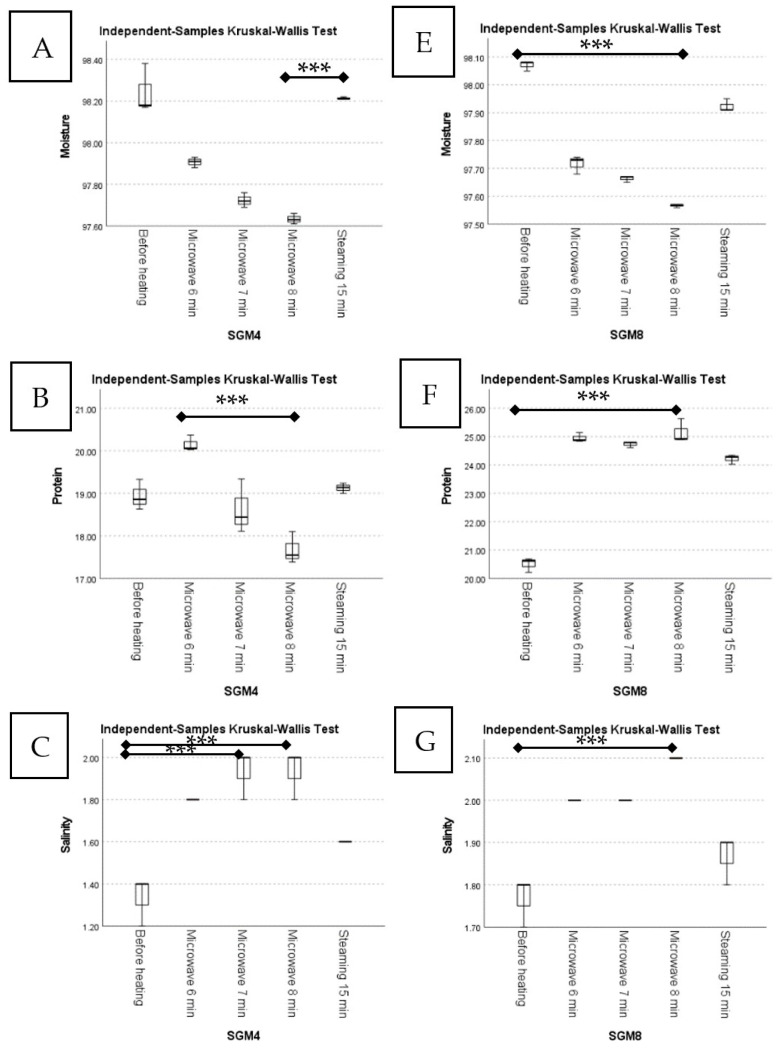

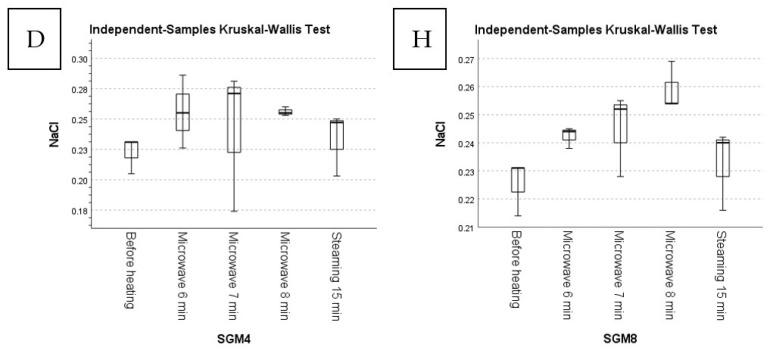
Boxplots of contents of (**A**) moisture, (**B**) protein, (**C**) salinity, and (**D**) sodium chloride of SGM4 before and after heat treatments and boxplots of contents of (**E**) moisture, (**F**) protein, (**G**) salinity, and (**H**) sodium chloride of SGM8 before and after heat treatments. *** between pairs indicate significantly different, according to Kruskal–Wallis and Dunn–Bonferroni tests (*p* ≤ 0.05).

For both SGM4 and SGM8, steaming under high pressure (autoclaving) caused less moisture loss than microwave heating. In fact, extending the time for microwave heating resulted in a higher loss of moisture due to increased evaporation [40,41]. Figure 8A,E show the findings of the Kruskal–Wallis test, which revealed that microwave heating for 8 min caused the highest moisture loss compared to steaming for the SGM4 sample (*p* = 0.026) and before heating in the SGM8 sample (*p* = 0.010). Microwave heating at 900 W for 6 min produced a higher protein content than the other heating conditions and was significantly different from microwave heating for 8 min in the SGM4 sample (Figure 8B; *p* = 0.010), which could be attributed to volumetric heating causing rapid heat transfer, allowing the protein to unfold faster and increasing the rate of protein digestion, with a subsequent increase in the nitrogen (and thus the protein content) of foods [42]. However, the longer the sample was microwaved, the faster protein hydrolysis occurred, resulting in more nutrients being lost due to amino acid degradation and the Maillard process [43,44]. Nonetheless, in the SGM8 sample, microwave heating for 8 min produced a higher protein content than before heating (Figure 8F; *p* = 0.019), which could be attributed to the extra protein substrate in SGM4 contributing to a balanced condition between protein digestion and degradation even after 6 min of microwaving. Furthermore, due to steric hindrance from dextran, microwave-assisted glucan glycosylation with proteins may reduce the change in the secondary structure of proteins, resulting in less excessive protein denaturation [45,46]. Furthermore, Thoresen et al. [47] found that prolonged microwave treatment caused protein aggregation, resulting in less available substrate for the enzymatic hydrolysis of proteins and thus slowing the breakdown process. 

In the current study, additional heating did not cause any changes in the NaCl content in either the SGM4 or SGM8 samples (Figure 8D,H, respectively; *p* > 0.05). On the other hand, microwave heating for 8 min increased salinity in both the SGM4 (Figure 8C; *p* = 0.047) and SGM8 (Figure 8G; *p* = 0.009) samples compared to those before heating, possibly because the microwaves induced a total breakdown of plant cell walls, increasing Na releasability within SGM [45,48]. This was consistent with another study reporting that microwaves could enhance the mineral contents of food [49].

**Figure 9:**
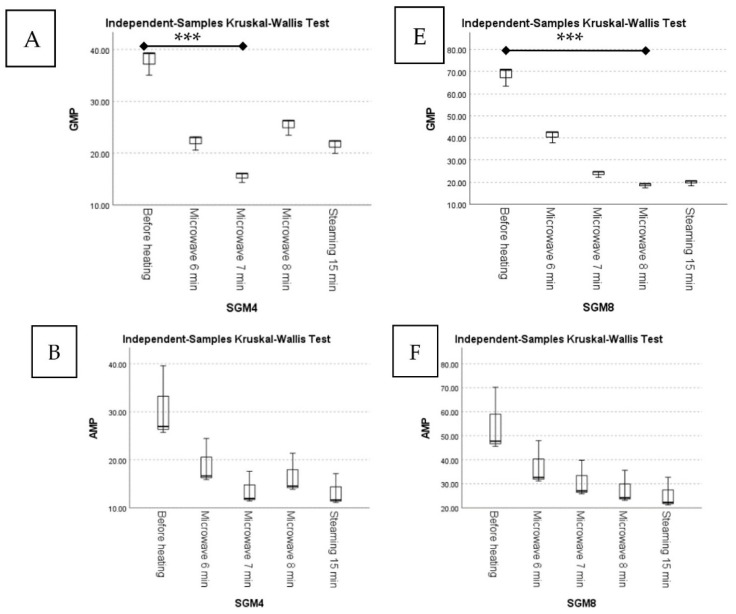

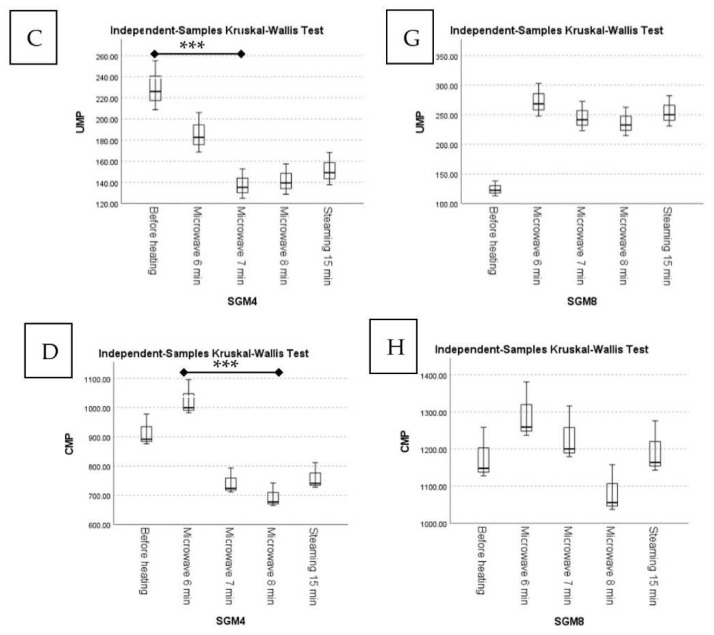
Boxplots of (**A**) 5′-GMP, (**B**) 5′-AMP, (**C**) 5′-UMP, and (**D**) 5′-CMP of SGM4 before and after heat treatments and boxplots of (**E**) 5′-GMP, (**F**) 5′-AMP, (**G**) 5′-UMP, and (**H**) 5′-CMP of SGM8 before and after heat treatments. *** between each pair means significantly different, according to Kruskal–Wallis and Dunn–Bonferroni tests (*p* ≤ 0.05).

According to Table 2, the mean loss of flavor nucleotides from autoclave steaming was greater than from microwave heating for a brief duration; however, the mean ranks were not significantly different (Figure 9; *p* > 0.05). Microwave treatment for 7–8 min resulted in greater 5′-GMP losses in both the SGM4 (Figure 9A; *p* = 0.010) and SGM8 (Figure 9E; *p* = 0.019) samples. The mean ranks of 5′-AMP before and after heat treatment were not significantly different, χ2(4) = 8.700, *p* = 0.069 in SGM4 (Figure 9B) and χ2(4) = 7.967, *p* = 0.093 in SGM8 (Figure 9F). In SGM4, microwave heating for 7 min significantly decreased 5′-UMP compared to the level before heating (Figure 9C; *p* = 0.047), but its effect was reversed in SGM8 (Figure 9G; *p* > 0.05). Microwave heating for 6 min increased the 5′-CMP content in SGM4, whereas microwave heating for 8 min decreased it substantially (Figure 9D; *p* = 0.035). In SGM8, the content of 5′-CMP increased with extra heating, whereas it decreased with a longer processing time. Nonetheless, the mean ranks did not vary significantly between groups, χ2(4) = 7.800, *p* = 0.099 (Figure 9H). It is possible that the microwave heating time had no significant impact on the concentration of 5′-nucleotides.

These findings were consistent with other studies reporting that thermal treatment facilitated and increased the release of certain compounds and their subsequent chemical or enzymatic reactions [36,37,39]. Heating caused thermal degradation and chemical reactions, such as enzymatic hydrolysis, the Maillard reaction, or Strecker degradation, all of which alter the amounts of sugars, amino acids, polyphenols, proteins, and carbohydrates [19]. Volatile and nonvolatile intermediates of thermal reactions contributed to flavor formation. Cooking white and cremini mushrooms at 163 °C for 10 min resulted in the loss of free amino acids and 5′-nucleotides [39], whereas sous vide cooking at 70 °C for 10 min resulted in the highest concentration of 5′nucleotides [36]. Temperature and time of extraction were discovered to be critical in the efficient extraction of umami compounds from mushrooms. Enzymatic hydrolysis of ribonucleic acids and oligonucleotides to form 5′-mononucleotides involved a minimum temperature of 70 °C, which could cause cell structure damage and allow molecules within the cells to escape into the extraction medium to form nucleotides. In contrast, because of temperature-assisted degradation and/or the association of extracted amino acids with other molecules present in the extract, free amino acids were extracted well at room temperature [50]. According to the findings of Li, Feng, Zhou, Zhou, Liu, Li, Ye, and Yang [37], higher temperatures and a longer duration caused greater degradation of extracted nucleotides, as evidenced in the current results by the lower levels of nucleotides in the microwave samples at 7–8 min compared to the microwaved sample at 6 min. Microwave heating is a volumetric heating technique resulting in a shorter heating time. For 5′-CMP and 5′-UMP, during the first few minutes of microwave heating, the nucleosides were released through the degradation of deoxyribonucleic acid or ribonucleic acid more than their loss, resulting in higher contents in the early stage, which then decreased as time passed [41,51,52]. Furthermore, microwave radiation accelerates the movement and collision of protein molecules, increasing the likelihood of interactions between the substrate protein and the enzyme and, as a result, accelerating the protein and amino acid degradation [53]. Other nucleotides might also follow this pathway, but to a lesser extent, as their initial contents were much lower.

Although not significantly different (*p* > 0.05), the majority of free amino acids in the standard soup with 0.4% SGM after microwave heating for 6 min were at higher levels than for before heating (Table 3), whereas the loss of free amino acids was greater in autoclaved samples than in the other samples.

Surprisingly, tyrosine was not detected in any of the samples except in SGM4 after 6 min of microwave heating, perhaps because microwave heating, in contrast to traditional heating, uses a combination of thermal and non-thermal effects to alter complicated protein structures by disrupting intramolecular interactions, which induced protein rotation and unfolding as well as the release of encrypted peptides triggered by successive hydrolysis and exposing aromatic amino acids, such as phenylalanine, histidine, and tyrosine [53]. In addition, when polar protein molecules collide, free radicals could be generated, resulting in the dissolution of disulfide bonds and the formation of sulfhydryl groups. As the total sulfhydryl group content grows, so does the quantity of hydrophobic, non-polar amino acids found in the interior of proteins. Tyrosine, as an amino acid with aromatic residues, can donate protons to electron-deficient radicals, allowing them to retain a high redox potential and contribute to antioxidant activity by stabilizing reactive molecules [45,54,55]. The most abundant amino acids in all samples were glutamic acid, aspartic acid, and arginine. The total amount of free amino acids decreased and increased in proportion to the accumulation caused by their consumption and production, just like for the 5′-nucleotides. Because of its ability to accelerate protein hydrolysis by volumetric heating, microwave treatment increased the content of free amino acids in the clear soup, which was consistent with the findings from other studies that reported higher levels of amino acids in microwaved samples compared to other heating methods, such as convection heating [41,42,44]. The EUC values of the flavor components in mushrooms can be divided into four categories: (1) >1000 g MSG/100 g dry weight, (2) 100–1000 g MSG/100 g dry weight, (3) 10–<100 g MSG/100 g, and (4) <10 g MSG/100 g [56]. SGM4 before heating had an EUC of 149.85 g MSG/100 g dry weight, whereas microwave heating and steaming under high pressure reduced the EUC by 43.11 and 44.53%, respectively, indicating that excessive heating influenced taste compounds.

## 4. Conclusions

In unseasoned clear soup samples, split-gill mushroom (SGM) powder at 0.2–0.8% concentrations had distinct taste profiles similar to salt, whereas samples with 0.2–0.8% concentrations had similar taste profiles to 0.4–0.6% MSG. Likewise, in seasoned soup samples, SGM at 0.4–0.8% had the same flavor-enhancing impact as 0.4% MSG. Additional microwave heating could increase the salinity of the clear soup due to the microwave-induced total disintegration of plant cell walls, which enhanced the release of metal ions, such as Na^+^. The effects of microwaves on protein, amino acids, and taste compounds could be explained as: (1) Microwaves cause polar molecules to clash, resulting in the formation of free radicals. These radicals damage disulfide bonds and encourage the breakdown of non-covalent bonds in the protein molecules, causing protein unfolding and conformational changes. (2) The exposed hydrophobic core residue of the depolymerized protein may make specific protein locations more susceptible to enzymatic hydrolysis, resulting in a smaller particle size, greater surface area, and more cleavage sites for digesting protease activity and hence increasing the protein content in the soup. (3) Excessive heating temperature and time cause proteins to aggregate into bigger molecular weight aggregates via hydrophobic and electrostatic cross-linking reactions, making protein endonuclease more difficult to reach and inhibiting its digestion and release. (4) At the same time, the protein expands under high-power microwave treatment, increasing the likelihood of effective collision with sugar molecules and enhancing the Maillard reaction, which has an inhibitory effect on proteolytic enzymes and can reduce digestibility via a mechanism similar to cross-linking aggregation between proteins. On the other hand, the Maillard reaction may promote an increase in hydrophobic and sulfur-containing amino acids, which can enhance the flavor of soup. Although there was no significant difference, it was observed that temperature and substrate concentration positively affected protein generation in both the microwave and conventional treatments; however, time had a more negative effect on the protein content of the microwaved samples than the conventional ones. Excessive heating could further reduce the amount of umami nucleotides and amino acids in both the microwave and conventional treatments, which lowered the EUC by 43.14% and 44.53%, respectively. Due to the limited number of replications, this study was unable to detect a significant difference between the conventional and microwave heating techniques, although increasing and decreasing trends from the means could be observed, which should be further investigated. In conclusion, using split-gill mushroom powder and microwave heating could be an alternative method for enhancing saltiness and umami tastes while potentially reducing the use of salt in clear soup; however, optimal microwave heating conditions should be further investigated to maximize the benefit of salty and umami taste perception enhancement.

## Figures and Tables

**Table 1:** Moisture, protein, and sodium contents of SGM4 and SGM8 before and after heat treatments.

Samples		Composition							
		Moisture (%wb.)	Mean Rank	Protein (%db.)	Mean Rank	NaCl (%wb.)	Mean Rank	Salinity (%wb.)	Mean Rank
SGM4	Before heating	98.24 ± 0.12	12.00 ^ab^	18.94 ± 0.36	8.00 ^ab^	0.22 ± 0.01	4.17 ^a^	1.30 ± 0.14	2.00 ^b^
	Microwave 6 min	97.91 ± 0.03	8.00 ^ab^	20.15 ± 0.19	14.00 ^a^	0.26 ± 0.03	10.33 ^a^	1.80 ± 0.00	9.00 ^ab^
	Microwave 7 min	97.72 ± 0.03	5.00 ^ab^	18.63 ± 0.64	7.00 ^ab^	0.24 ± 0.06	9.33 ^a^	1.93 ± 0.11	12.00 ^a^
	Microwave 8 min	97.63 ± 0.02	2.00 ^b^	17.68 ± 0.38	2.00 ^b^	0.26 ± 0.00	10.00 ^a^	1.93 ± 0.11	12.00 ^a^
	Steaming 15 min	98.21 ± 0.00	13.00 ^a^	19.13 ± 0.12	9.00 ^ab^	0.23 ± 0.03	6.17 ^a^	1.60 ± 0.00	5.00 ^ab^
SGM8	Before heating	98.07 ± 0.02	14.00 ^a^	20.51 ± 0.25	2.00 ^b^	0.23 ± 0.01	3.33 ^b^	1.77 ± 0.06	2.33 ^b^
	Microwave 6 min	97.72 ± 0.03	8.00 ^ab^	24.95 ± 0.16	11.67 ^ab^	0.24 ± 0.00	8.33 ^ab^	2.00 ± 0.00	9.50 ^ab^
	Microwave 7 min	97.66 ± 0.01	5.00 ^ab^	24.73 ± 0.10	8.00 ^ab^	0.24 ± 0.01	9.33 ^ab^	2.00 ± 0.00	9.50 ^ab^
	Microwave 8 min	97.57 ± 0.01	2.00 ^b^	25.15 ± 0.42	13.33 ^a^	0.26 ± 0.01	13.33 ^a^	2.10 ± 0.00	14.00 ^a^
	Steaming 15 min	97.92 ± 0.02	11.00 ^ab^	24.22 ± 0.16	5.00 ^ab^	0.23 ± 0.01	5.67 ^ab^	1.85 ± 0.07	4.67 ^ab^

Data presented as mean ± standard deviation and mean rank, where ^a,b^ = mean rank values within column followed by different lowercase superscripts are significantly different, according to Kruskal–Wallis and Dunn–Bonferroni tests (*p* ≤ 0.05).

**Table 2:** 5′-Nucleotides of SGM4 and SGM8 before and after heat treatments.

Samples		5′-Nucleotides (mg/100 g; Dry Weight)										
		5′-GMP	Mean Rank	5′-AMP	Mean Rank	5′-IMP	Mean Rank	5′-UMP	Mean Rank	5′-CMP	Mean Rank	Total Nucleotides
SGM4	Before heating	37.89 ± 2.45	14.00 ^a^	30.73 ± 7.68	14.00 ^a^	n.d.	-	229.98 ± 23.39	14.00 ^a^	915.12 ± 54.72	11.00 ^ab^	1213.72
	Microwave 6 min	22.25 ± 1.44	7.33 ^ab^	18.99 ± 4.75	9.00 ^a^	n.d.	-	185.83 ± 18.90	11.00 ^ab^	1025.71 ± 61.33	14.00 ^a^	1252.78
	Microwave 7 min	15.51 ± 1.00	2.00 ^b^	13.67 ± 3.42	5.33 ^a^	n.d.	-	137.70 ± 14.00	3.67 ^b^	742.75 ± 44.41	5.00 ^ab^	909.63
	Microwave 8 min	25.37 ± 1.64	11.00 ^ab^	16.59 ± 4.15	7.33 ^a^	n.d.	-	141.89 ± 14.43	5.00 ^ab^	694.53 ± 41.53	3.33 ^b^	878.38
	Steaming 15 min	21.57 ± 1.39	5.67 ^ab^	13.31 ± 3.33	4.33 ^a^	n.d.	-	151.73 ± 15.43	6.33 ^ab^	759.78 ± 45.43	6.67 ^ab^	946.39
SGM8	Before heating	68.44 ±4.42	14.00 ^a^	54.53 ± 13.63	13.33 ^a^	n.d.	-	124.46 ± 12.66	2.00 ^a^	1177.94 ± 70.44	6.33 ^a^	1425.37
	Microwave 6 min	41.01 ± 2.65	11.00 ^ab^	37.29 ± 9.32	9.67 ^a^	n.d.	-	273.05 ± 27.77	12.00 ^a^	1292.04 ± 77.26	12.33 ^a^	1643.39
	Microwave 7 min	23.88 ± 1.54	8.00 ^ab^	30.93 ± 7.73	7.33 ^a^	n.d.	-	245.70 ± 24.99	8.67 ^a^	1231.61 ± 73.65	10.33 ^a^	1532.12
	Microwave 8 min	18.58 ± 1.20	2.67 ^b^	27.67 ± 6.92	5.67 ^a^	n.d.	-	236.73 ± 24.07	7.33 ^a^	1083.39 ± 64.78	3.00 ^a^	1366.37
	Steaming 15 min	19.82 ± 1.28	4.33 ^ab^	25.40 ± 6.35	4.00 ^a^	n.d.	-	254.49 ± 25.88	10.00 ^a^	1194.55 ± 71.43	8.00 ^a^	1494.26

Data presented as mean ± standard deviation and mean rank, where ^a,b^ = mean rank values within column followed by different lowercase superscripts are significantly different, according to Kruskal–Wallis and Dunn–Bonferroni tests (*p* ≤ 0.05), and n.d. = not detected.

**Table 3:** Free amino acids of SGM4 before and after heat treatments.

Amino Acid (mg/100 g; Dry Weight)	SGM4				
	Before Heating	Microwave 6 min	Steaming 15 min	χ2 (2) ^#^	*p*-Value
Glutamic acid	1269.34 ± 21.59	1216.45 ± 5.33	1249.25 ± 28.96	3.714	0.156
Aspartic acid	602.34 ± 0.12	607.31 ± 0.19	552.75 ± 15.15	4.571	0.102
Threonine	208.83 ± 2.28	223.70 ± 0.62	201.71 ± 3.83	4.571	0.102
Serine	238.07 ± 6.26	255.63 ± 0.45	229.78 ± 3.92	4.571	0.102
Glycine	265.78 ± 4.02	280.13 ± 1.50	258.67 ± 10.90	3.714	0.156
Alanine	332.14 ± 12.02	339.73 ± 0.44	320.38 ± 9.13	2.000	0.368
Cystine	n.d.	n.d.	n.d.	-	-
Valine	233.88 ± 6.17	264.83 ± 4.64	240.78 ± 0.83	4.571	0.102
Methionine	n.d.	n.d.	n.d.	-	-
Isoleucine	163.54 ± 1.38	176.80 ± 3.16	160.62 ± 2.48	4.571	0.102
Leucine	316.23 ± 0.99	342.03 ± 0.81	307.64 ± 8.43	4.571	0.102
Tyrosine	n.d.	<250	n.d.	5.000	0.082
Phenylalanine	<250	<250	<250	-	-
Histidine	105.82 ± 8.04	110.28 ± 0.47	<100	3.529	0.171
Hydroxylysine	n.d.	n.d.	n.d.	-	-
Lysine	207.22 ± 7.71	194.52 ± 6.70	153.90 ± 9.08	4.571	0.102
Arginine	687.21 ± 12.52	622.56 ± 2.97	438.99 ± 9.89	4.571	0.102
Hydroxyproline	n.d.	n.d.	n.d.	-	-
Proline	201.57 ± 3.63	222.39 ± 5.79	<200	4.194	0.123
Tryptophan	<150	<150	<150	-	-
EUC (g/100 g)	149.85	85.25	83.12	-	-

Data presented as mean ± standard deviation, χ2, and *p*-value of Kruskal–Wallis H test. n.d. = not detected. ^#^ The number presented n of samples-1.

## Data Availability

The data in this study are available in the article.
